# Effect of Surgical Outcome on the Psychologic Aspect of Pain

**DOI:** 10.5812/kowsar.22287523.1990

**Published:** 2011-09-26

**Authors:** Charu Mahajan, Girija Prasad Rath

**Affiliations:** 1 Department of Neuroanaesthesiology, India Institute of Medical Sciences, New Delhi, India

**Keywords:** Depression, Back pain, Discectomy

*Dear Editor*,

We read with great interest the recent article by Farzanegan et al. regarding the effect of lumbar discectomy on disability and depression in patients with chronic low back pain (1). Pain and depression create a vicious cycle in which pain worsens the symptoms of depression, and then the resultant depression further worsens the feeling of pain. The authors maintain that lumbar discectomy breaks this cycle, which is indeed an interesting possibility. However, it is unclear whether or not patients with a family history of depression and those on antidepressants were excluded from the study. France et al. suggested that the occurrence of major depression in patients with chronic back pain might be related to a genetic vulnerability to depression in this group of patients (2). Recurrent disc herniation is one of the most important reasons for unsatisfactory results, and consequently failed back syndrome. The recurrence rate of lumbar disc herniation has been reported in 5%–12% of patients after surgery (3). Thus, the follow-up period should be sufficiently long to detect any recurrence and its effect on patient psychology. Only then will the effect of intervention on the quality of life be satisfactorily addressed. Psychosocial factors have a significant influence on pain perception. Arpino et al. demonstrated the negative role of depression on outcome after lumbar disc surgery. They emphasized the need to consider psychological factors in such patients (4). The influence of these confounding factors may partially explain why surgery is not always successful (5). Thus, the relationship between depression and surgical outcome may be considerably more complicated than is generally believed. Nevertheless, research should be aimed at improving the quality of life of our social strata, and the authors’ initiative in taking such a step forward is commendable.
